# Kentucky State Dental Society

**Published:** 1871-12

**Authors:** 


### KENTUCKY STATE DENTAL SOCIETY.

It was our good fortune, recently, to attend a meeting of this Society, held at Louisville, Ky. The spirit and energy there manifested were very gratifying, indeed. The principal work before the meeting at that time was the establishment of a dental infirmary in the city of Louisville, the object of which is two-fold, viz.: to afford facilities for general consult tion, comparison, and clinics, and to give to the poor the advantages of treatment and operations upon their teeth, which they can not have in the private office. There should be such an institution established and sustained by the profession in every city and large town in the country. The plan decided upon here contemplates the procurement of a person competent to take charge, under the proper committee, of the institution, and keep it open at least a portion of every day for the admission and treatment of patients. The members of the Society will, by appointment, be present for duty at stated periods. The practice of the infirmary may be made, and doubtless will be, very valuable to the junior members of the profession. Regular, systematic infirmary facilities, and clinic practice, outside of the Dental Colleges, in Brooklyn, New York and Louisville: who next?

We are just informed that the infirmary at Louisville is in active operation, as contemplated.

